# Understanding the subjective experiences of memory concern and MCI diagnosis: A scoping review

**DOI:** 10.1177/14713012221147710

**Published:** 2022-12-27

**Authors:** Christine Carter, Tiffeny James, Paul Higgs, Claudia Cooper, Penny Rapaport

**Affiliations:** Division of Psychiatry, 4919 University College London, London, UK; Division of Psychiatry, 4919 University College London, London, UK; Division of Psychiatry, 4919 University College London, London, UK; Division of Psychiatry, 4919 University College London, London, UK; Division of Psychiatry, 4919 University College London, London, UK

**Keywords:** Memory concerns, mild cognitive impairment, liminality, subjective experiences, diagnosis, age, agency, active ageing, relationships, roles

## Abstract

**Introduction:**

Many older people experience memory concerns; a minority receive a diagnosis of Mild Cognitive Impairment (MCI) or Subjective Cognitive decline (SCD). There are concerns that medicalisation of MCI and memory concern may fail to acknowledge subjective experiences.

**Aim:**

We explore the meaning individuals give to their memory concerns, with or without a diagnosis of MCI and SCD.

**Method:**

We scoped literature exploring subjective experiences of memory concern, with or without a diagnosis of MCI or SCD. We searched CINAHL, PsycINFO and MEDLINE in March 2020, and updated in Sept 2021.We used (Arksey & O’Malley, 2005) framework to guide our scoping review method and thematic analysis to analyse our findings.

**Results:**

We screened 12,033 search results reviewing the full texts of 92 papers. We included 24 papers, including a total of 453 participants, the majority of whom were female, from White ethnic majority populations (or from studies where ethnicity was not identified) with high levels of education. In 15 out of 24 studies, 272 participants were diagnosed with MCI. We identified two themes; *Making a diagnosis personal* and *Remembering not to forget*. We found that subjective experiences include normative comparison with others of the same age and responses including fear, relief, and acceptance, but culminating in uncertainty.

**Conclusion:**

Drawing upon sociology, we highlight the subjective experiences of living with memory concerns, SCD and an MCI diagnosis. We identify a gap between the intended purpose of diagnostic labels to bring understanding and certainty and the lived experiences of those ascribed them.

## Introduction

Many older people have concerns about their cognitive function, which generate uncertainty and confusion. Some seek help and receive a diagnostic label. Mild Cognitive Impairment (MCI) and Subjective Cognitive Decline (SCD) are diagnostic categories which describe respectively, objective, and subjective cognitive concerns judged by the diagnostician to be inconsistent with age, but not indicative of dementia.

There is ambiguity around the diagnosis of MCI (Schweda et al., 2018) despite its inclusion in the Diagnostical Statistical Manual-5 (DSM-5). Doubts regarding the value and utility of the diagnosis create dilemmas for those conferring, and in receipt of, this diagnostic ‘label’. The conversion rate from MCI to dementia varies. McGrattan et al., (2022) study found a range of 6.0%–44.8%; with an average follow-up 3.7 years. SCD is sometimes a precursor to MCI. Opdebeeck et al., (2019) found limited associations between SCD and objective cognition, querying the worth of this term as a category (Gifford et al., 2015). Most individuals meeting the criteria for MCI and SCD are undiagnosed, so these labels are descriptors of the minority of people who seek help for mild memory concerns.

Literature focuses upon prognostic implications of MCI/SCD, with less attention given to the subjective experiences of diagnosis, and memory concerns (Gerstenecker and Mast (2015)). (Beard & Neary, 2013) describe how the medicalisation of MCI and memory concern does not account for the subjective accounts of lived experiences of the condition. Our scoping review is the first, to our knowledge, to explore literature on individual experiences of living with memory concern that is not dementia. Scoping reviews explore and understand knowledge in an emerging field and discuss characteristics in that field (Peters et al., 2020). We draw upon notions of liminality, agency, and sociological concepts of age through the third and fourth age to explore our review findings.

## Methods

Our review was guided by (Arksey & O’Malley, 2005) 6 stage framework for scoping reviews and Levac and colleagues (2010) paper on advancing scoping review methodology. We also incorporated contemporary guidance from the Joanna Brigg Institute (JBI) which points to scoping reviews as being mostly descriptive, with authors having the flexibility to undertake more in-depth analyses to inform research questions. We used thematic analysis techniques to conduct a qualitative meta-synthesis of our findings. We report our methods using headings from Arksey and O’Malley’s (2005) framework,

Stage 1) Identifying the research questions

To capture the individuality of lived experience, we included subjective terminology in our research questions

➢ What meaning do individuals give to their memory concerns, including those with or without a diagnosis of MCI and SCD.
➢ How do they understand this in context of cognitive impairment and ageing
Stage 2) Identifying relevant studies

We searched CINAHL, PsycINFO and MEDLINE on 12/3/2020 for words relating to three concepts. Concept 1 included search terms around MCI, subjective cognitive decline, memory concerns and related terms used within a public domain. Concept 2 included words relating to older people, age and later life. Concept 3 included words for subjective experiences such as feelings, and meaning. A full search example is included in Appendix 1. We also hand searched reference lists of relevant articles. We re-ran searches in September 2021.

Stage 3) Study selection: Inclusion criteria

**Figure 1.**
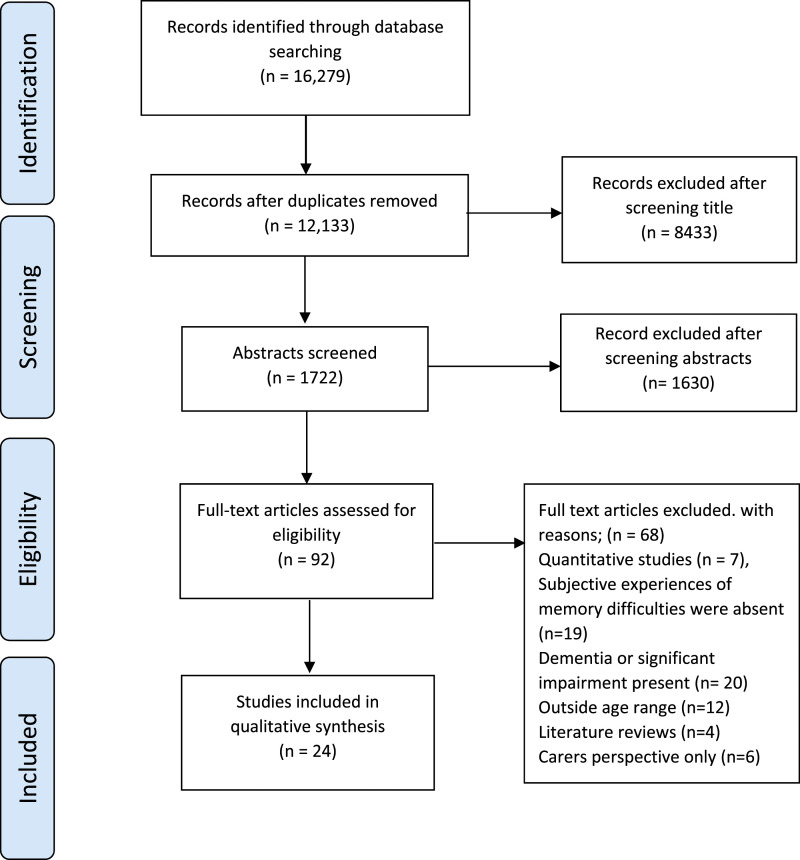
PRISMA flow diagram of screening process.

We included primary qualitative or mixed methods research studies, published in English in peer reviewed journals, reporting the experiences of populations, all of whom were aged 55 and over and living with a cognitive impairment which was not dementia e.g., MCI, SCD or other memory concerns. We excluded review articles but individual papers on review reference lists were considered for inclusion. Following de-duplication, search results were screened via title by one researcher (CC), excluding any that were irrelevant. The remaining were screened by abstract with a second researcher (PR) double screening 20% to standardise the screening process. We resolved any disagreements through discussion. This process resulted in 92 articles for full text screening. Papers were critically discussed with reviewer (PR) and a second blinded reviewer (TJ) reviewed a sample of six selected papers in depth. The PRISMA flow diagram in Figure 1 shows the screening process.

Stage 4) Charting the data

We (CC, PR & TJ) produced a data extraction table (Appendix 2) and extracted, for each paper: country of publication, research aims, sample population, data collection method, data analytic approach, and key themes identified. We used the Critical Appraisal Skills Programme tool for qualitative studies (CASP 2019). Studies were not excluded on the grounds of quality, in line with qualitative review practice (Thomas & Harden, 2008). The quality assessment table is in Appendix 3.

Stage 5) Collating, summarising, and reporting the results

Full papers were imported into NVivo 11 for thematic synthesis (Braun & Clarke, 2021). Where papers were mixed methods only qualitative results were used. We were guided by Thomas and Harden’s (2008) approach to thematic synthesis. Primary qualitative data included within the published papers, the reported themes and supporting quotes were coded. Using Nvivo we organised and categorised papers themes as codes. (CC) coded data guided by our research questions and (PR) and (PH) reviewed this data. Quotes from participants were used to preserve their voice and retain the integrity of the original studies, recommended in scoping reviews (Peters et al, 2020). CC re-read papers and developed overarching themes, further synthesis of themes was achieved through discussion within the co-author group.

Stage 6) Consultation stage

Consultation is a recommended part of the scoping review process. (Arksey & O’ Malley 2006) We consulted on our findings with a Patient Participation Involvement (PPI) group from the APPLE-tree multi-domain health and lifestyle intervention for people aged 60 + who have memory concerns with or without a diagnosis of MCI (Cooper et al., 2020). We held the session on zoom with 3 people. All participants were female, one described herself as having ‘early stage Alzheimer’ the second cared for parents with memory problems and the third was the APPLE-tree programme manager. Using our two themes we presented quotes from selected studies within the literature to facilitate a discussion. Participants felt the quotes reflected their own personal experiences, describing them as ‘accurate and valid’ and another saying they were ‘spot on’.

## Findings

### Study selection

Our search yielded 16,279 studies, from which we included 24 papers (see Figure 1 for PRISMA).

### Country of origin

Seven studies took place in the UK (Corner & Bond, 2004; Dean et al., 2014; Giebel et al., 2016; Gomersall et al., 2017; Meilak et al., 2016; Pierce et al., 2016; Poppe et al., 2020), followed by five papers from the USA (Beard & Neary, 2013; Hill et al., 2018; Kruger TM, 2014; Lingler et al., 2006; Renn et al., 2021). Two papers were from Israel (Rotenberg et al., 2020; Werner, 2004) and two were from Canada (Parikh et al., 2016; Vandermorris et al., 2017). The remaining papers were from Belgium, Sweden, Holland, New Zealand, South Korea, and Japan.

### Participant characteristics

The 24 papers included a total of 211 male participants and 242 female participants with ages ranging from 55 to 93 years. Participants in 15 papers had a diagnosis of MCI, ranging from within the last 6 months (Rotenberg et al., 2020), to over 7 years ago (Berg et al., 2013). Seven papers included participants described as having memory concerns (subjective or objective), whilst in two papers participants were screened as having SCD.

From this point forward when we use the term ‘memory concerns’ we refer to people with varying degrees of cognitive impairment who experience this subjectively and objectively and which includes people with a diagnosis of MCI or screened for SCD and those without. When we just use the term ‘MCI’ we refer to papers where participants specifically have a diagnosis of MCI.

Out of the 24 papers selected only 15 reported on the ethnicity of the participants. Where ethnicity was documented the majority were reported as being Caucasian and white British, only 3 participants described themselves as African Caribbean British (Meilak et al., 2016; Poppe, 2020). One UK study focus was on South Asian participants described as Indian British and Pakistani British (Giebel et al., 2016). Studies from New Zealand described participants who identified as native New Zealand and the South Pacific Islands,(Collier et al., 2020) two papers were from Japan and Korea respectively, (Choi & Kim, 2020; Nakano et al., 2012) and one paper from Israel described European and Asian participants but didn’t specify further, (Werner, 2004). The majority of participants were reported as having moderate to high levels of education**.**

### Population and settings

Ten studies recruited from memory clinics,(Begum et al., 2013; Dean et al., 2014; Giebel et al., 2016; Gomersall et al., 2017; Imhof et al., 2006; Joosten-Weyn Banningh et al., 2008; Lingler et al., 2006; Meilak et al., 2016; Pierce et al., 2016; Poppe et al., 2020); seven recruited sub-samples from larger studies, (Beard & Neary, 2013; Berg et al., 2013; Collier et al., 2020; Corner & Bond, 2004; Kruger TM, 2014; Rotenberg et al., 2020; Vandermorris et al., 2017). Five papers recruited from community settings (Begum et al., 2013; Hill et al., 2018; Nakano et al., 2012; Poppe et al., 2020; Werner, 2004), defined as rural, urban, community dwelling and third sector community organisations, four papers purposively recruited from Geriatric Day/Hospitals settings (Choi & Kim, 2020; De Vriendt et al., 2012; Parikh et al., 2016; Renn et al., 2021).

### Thematic analysis

Thematic analysis generated two themes; *Making a diagnosis personal* and *Remembering not to forget*. Themes and subthemes reflect both the breadth of the literature and commonalities within in.

### Making a diagnosis personal

This theme describes how individuals respond to MCI and how a lack of clear knowledge surrounding memory concerns creates feelings of uncertainty. We identified 3 sub-themes. These explore; (1) the uncertainty and reactions surrounding a diagnosis of MCI due to a lack of knowledge, (2) the normalisation of the experience of memory concern with others of a similar age and (3) Coming to terms with accepting MCI *.* People either appear to accept these experiences, some that feel frightened by the uncertainty and others need to fill in the gaps by learning more.

#### Sub-theme 1: Responding to uncertainty and certainty of a diagnosis.

The lack of clarity around a diagnosis of MCI results in feelings of uncertainty, (Beard & Neary, 2013; Gomersall et al., 2017; Poppe, 2020). The impact of this is exemplified by a participant diagnosed with MCI, who expressed uncertainty and questioned the diagnosis several times during an interview,

*‘‘I don’t know how important [an MCI diagnosis] is, or not important. Is it very important? Seriously important? Or not very important? I don’t know’’* (Collier et al., 2020 p.306)

In attempting to gain clarity participants sought knowledge around the physical nature of memory impairment (Choi & Kim, 2020; Collier et al., 2020; Giebel et al., 2016; Imhof et al., 2006; Renn et al., 2021). Collier et al., (2020) describes how following a diagnosis of MCI, a participant learnt about the physical structure and function of the brain. Reactions and responses to MCI include, relief, acceptance, distance and fear. (Beard & Neary, 2013; Begum et al., 2013; Berg et al., 2013; Corner & Bond, 2004; Gomersall et al., 2017; Kruger TM, 2014; Lingler et al., 2006; Pierce et al., 2016; Rotenberg et al., 2020). Relief was expressed when finding out the diagnosis was MCI and not Alzheimer’s disease. A participant who was 7 years post MCI diagnosis comments,

*“when I spoke to the doctor after the investigation I was told that it was not Alzheimer I had and it was so wonderful to hear that”* (Berg et al., 2013 p.296)

However, ‘a new’ diagnosis of MCI, ‘downgraded’ from Alzheimer’s may fail to assuage worry. In the following example a participant who had been given a diagnosis of MCI after previously being told that she had Alzheimer’s said the following after reading a leaflet about MCI,

*“I thought: I haven’t got it, well, what have I got then? And I were reading that and I thought: what’s this? This is more or less saying a similar thing to er ... dementia”* (Gomersall et al., 2017 p.793)

These experiences illustrate how in practice, the concept of MCI lacks clarity. Being given a diagnosis of MCI allowed individuals to distance themselves from dementia yet the uncertainty of developing dementia is not eliminated., (Beard & Neary, 2013; Begum et al., 2013; Lingler et al., 2006). A participant with memory concern who had a close relative with Alzheimer’s describes the fear this induced,

*“and this is one thing I do just dread, and that is Alzheimer’s, very much so, because my father had it...the mere thought of dementia terrifies me...more than any other thing [disease] to die from ... like cancer”* (Corner & Bond, 2004 p.147)

Corner & Bond, (2004) interviewed people categorised ‘at risk’ of memory impairment, finding concerns about dementia increased where a relative had developed it, and the perception was that little could be done by contacting health professionals,

*“There’s not much point going to your doctor, really is there, I mean it’s not like they can cure it or anything”* (Corner & Bond, 2004 p.149)

Linglers (et al. 2006) study supports this and reports that participants preferred to construct personal narratives about their diagnosis with only sporadic references to health care professionals as a source of information. Giebel et al (2016) found that those who had not consulted a GP about their memory concerns were more likely to describe themselves as leaving things to fate.

#### Sub-theme 2: Normalising memory concerns through age and shared experiences

Normalising memory concerns through age and ageing is well documented in the literature, with uncertainty and disagreement among experts how best to characterize the relationship between ageing and dementia (Beard & Neary, 2013; Begum et al., 2013; Corner & Bond, 2004; Giebel et al., 2016; Kruger TM, 2014; Parikh et al., 2016; Renn et al., 2021; Vandermorris et al., 2017). Within this context, individuals must navigate the meanings around MCI, diagnosis and their relationship to age. Imhof, (2006) and Renn, (2021) both discuss how age is presented as an explanation of memory concerns which can be reassuring and how disclosure of this to others is a source of support, illustrated here from a participant diagnosed with MCI;

*“and we talk to each other because we’re all more or less in the same boat”* (Begum et al., 2013 p.468)

Expectations about age could distance individuals from services, limiting support or invalidating concerns, (Dean et al., 2014; Gomersall et al., 2017; Imhof et al., 2006; Renn et al., 2021). Illustrated by a female participant diagnosed with MCI who, on seeing her GP about memory concerns was asked;

*“have you considered your age Mrs? [She describes being furious, saying]; ‘absolute rubbish we go to church with elderly people in their mid and late nineties, maybe wobbly on their feet but as alert as anything”* (Dean et al., 2014 p.477)

Positioning herself in relation to older peers resulted in feeling this was not normal for her age. Individuals’ lack of clarity of how MCI differed from ‘normal’ ageing and dementia mirrors uncertainty within professional diagnosis; illustrated by a participant with MCI,

*“I don’t know what [MCI] means […] I want to know, I sort of need to know. I mean, with other people who are my age and my activity […] how does that fit alongside people of similar circumstances? I don’t know”* (Collier et al., 2020 p.307)

Shared activities and settings served to normalise changes in memory. For example, being in a senior living community made it easier to talk about memory decline as *‘people accept it because it’s not uncommon here’*, (Renn et al., 2021). In Renn’s study a participant with a diagnosis of MCI talked about giving up driving,

*“it’s not a premature thing a lot of 80 years old I know don’t drive”* (Renn et al., 2021 p.1102)

Vandermosis et al. (2017) and Beards (2013) studies suggest that normalising cognitive decline through age allows individuals to view memory concerns as evidence they don’t have a serious problem, such as Alzheimer disease

*“It’s not early stage Alzheimer’s. It’s not MCI. I have a situation where I can’t remember something. But, I don’t think that is different than other people my age”* (Beard & Neary, 2013 p.137)

Individuals re-frame the MCI label as part of normalising their memory problem, evident in the above quote by a participant diagnosed with MCI within the last 3 years,

#### Sub-theme 3: Coming to terms with accepting MCI

Time-based or temporal factors shape understandings of MCI. A study participant on receiving a diagnosis of MCI perceived a friend’s experience of memory decline as rapid and used this to position himself.

*“I know one guy who went from six months ago MCI to losing it all in 6 months so I’m aware how serious it is”* (Kruger, 2014 p.120)

Corner and Bond (2004) highlight a complex picture due to unknown trajectories of MCI raising questions for individuals. When asked about future expectations one participant with MCI said they didn’t feel there was any future, and at the same time reconciled this to themselves;

*“the future? Oh, the future is behind me! (laughs) You know, I have had a great life, I have had a good job and kids, and. . . all that is behind me you know. I just hope I will have some good and healthy years now”* (Berg et al., 2013 p.297)

Renn et al., (2021) describes how people with MCI can ‘seek a sense of calmness’, suggesting a more positive acceptance about their future life, One participant, for example described it as ‘*taking stock of life’*. The confusion about length of progression from MCI to something ‘worse’ is illustrated by this quote from a person living with MCI,

*“Well I do worry if it gets worse, ‘I don’t want to end up like a cabbage.* *You need your faculties don’t you in life”* (Pierce et al., 2016 p.8)

Although not using the term Alzheimer’s, perceptions of progression reflect a fear of losing control and an ability to function independently. The belief that ‘it could be worse’, means it could have been a diagnosis of dementia. An extract below from a participant with MCI demonstrates this,

*“I have to resign myself to it’’ and ‘‘I have to convince myself that they couldn’t find anything and accept that it’s not dementia I’m suffering from”* (Joosten-Weyn Banningh et al., 2008 p.152)

Many researchers suggest that individuals normalise their forgetfulness rather than actively fight it, (Beard & Neary, 2013; Joosten-Weyn Banningh et al., 2008; Parikh et al., 2016; Pierce et al., 2016). This is, illustrated in the following from a participant with an MCI diagnosis;

*“If I can’t do anything about it, I accept it, and that’s the way it is. That’s life”* (Parikh et al., 2016 p.881)

In the absence of clear information, it could be argued that MCI generates both acceptance and inevitability.

### Remembering not to forget

This theme describes actions individuals undertake adapting and responding to memory concerns, highlighting the emotional effort this involves. We identified 3 sub-themes, 1) attempts to make sense of memory concerns, 2) using everyday compensatory strategies, 3) how individuals navigate relationships with friends and family.

#### Sub-theme 1: The effort and impact of adapting actions and to stay active

Individuals respond to memory concerns by adapting which sometimes includes failing, with responses requiring tremendous effort (Corner & Bond, 2004; Hill et al., 2018; Meilak et al., 2016; Renn et al., 2021). Imhof et al. (2006) described participants as, *‘trying to manage failure and reorganizing tasks to reduce complexity’*. This is illustrated in the following extract which indicates how exhausting the conscious effort of remembering can be, this participant did not have a diagnosis of MCI but had concerns about their memory;

*“I need to think and keep my attention on what I am doing, and I am tired of thinking… In earlier days, I managed to do a thousand things at the same time”* (Imhof et al., 2006 p.350)

Adapting to the disruption caused by memory concerns, affirms ability to continue that activity, exemplified by the following participant who had memory problems for more than 6 months but no MCI diagnosis;

*“I started reading a book, and only about half way though I realized that I had already read it. For a while I almost stopped reading because of this. Eventually I got back to reading books, because it’s entertainment, and as long as it’s fun while I read, that’s enough for me”* (Rotenberg et al., 2020 p.6)

Individuals try to avoid fixating on the process of remembering, as illustrated in the following extract from a participant who has been screened has having SCD,

*“I try to make the things I need to remember less important”* (Hill et al., 2018 p.5)

This suggests a pressure to perform, linked to the concept of a *‘use it or lose it’* belief described by Meilak et al. (2016). Pressure to compensate through writing everything down also acts as a reminder of individual limitations in people concerned about their memory, illustrated in the following;

*“I have a diary there and I write everything down that I’ve got to do—all my appointments, dentist, everything. I think Oh God! I’m losing my marbles”* (Corner & Bond, 2004 p.5)

Renn, (2021) describes how ‘helpful’ strategies imposed by others could produce feelings of shame. A participant described how using a whiteboard calendar set up by his wife to plan his week left him feeling embarrassed. Thus, the intervention response inadvertently positions the individual as feeling they are the problem. There appears to be juxtaposition of creating and maintaining strategies to compensate for the impact of memory loss, yet simultaneously these serve as a constant reminder of that loss. Feelings of stupidity amongst participants in relation to MCI or memory concerns have been documented, (Berg et al., 2013; Collier et al., 2020; Gomersall et al., 2017; Nakano et al., 2012; Parikh et al., 2016). Emotional reactions ranged from bewilderment to feeling stupid over memory mistakes, as exemplified by a participant with a diagnosis of MCI,

*“If I say something and [my son] says, “Oh, you’ve said that before….” That destroys you when they say, “you’ve said that before”* (Parikh et al., 2016 p.881)

Feeling “destroyed” when memory mistakes are brought to his attention highlights the impact that this may have on self-esteem and confidence for some individuals, (Parikh et al., 2016).

#### Sub-theme 2: Using compensatory strategies everyday

Compensatory strategies minimise the disruption memory concerns create, such as writing lists, using diaries, creating routines, visualising travel routes and repetition of information, (Corner & Bond, 2004; Hill et al., 2018; Imhof et al., 2006; Joosten-Weyn Banningh et al., 2008; Meilak et al., 2016; Parikh et al., 2016; Renn et al., 2021). ‘Self–management strategies’ were undertaken as a way of compensating for memory concerns, (Begum et al., 2013; Gomersall et al., 2017). Parikh et al., (2016) study describes this as ‘*reliably remembering’*, a process of helping with “self-esteem” and “coping”, expressed by the following participant with MCI;

*“It’s just a matter of compensating, learning how to compensate…. I think we’ve adjusted so we can continue doing what we’ve done for years and years”* (Parikh et al., 2016 p.882)

However, studies describe individual’s inability to remember to use the external compensatory strategies they develop, exemplified in the following extract from a participant screened as having SCD;

*“Well, I try to associate other names or other ideas with it and sometimes it works, then other times, I figure I have an association, and then I can’t* figure *the association”* (Hill et al., 2018 p.7)

Individual’s previous careers supported compensatory strategies, such as using appointment books*.* (Berg et al., 2013; Hill et al., 2018; Parikh et al., 2016; Poppe, 2020).

#### Sub-theme 3: Relationships and connections with others

Poppe’s (2020) study found that maintaining a job and socialising were deemed important ways of staying connected, affecting views of retirement and activity. Personal relationships, such as spousal relationships frequently provided sources of practical and emotional support, (Begum et al., 2013; Dean et al., 2014). Partner relationships are significant in how individuals live and cope with memory concerns, a female participant with MCI illustrated this after being asked about support from publicly funded services

“*without my husband I wouldn’t have managed this well”* (Dean et al., 2014 p.481)

The importance of relational support through family and friends is well documented, (Collier et al., 2020; Dean et al., 2014; Parikh et al., 2016; Pierce et al., 2016; Renn et al., 2021). Research describes how friendships help participants with MCI cope with stress, as illustrated below;

*“I’ve got good friends and sometimes you just got to let it out, then I can calm down then I carry on”* (Dean et al., 2014 p.481)

Literature suggests relationships need to be adaptable to be supportive, examples of friends adjusting to an individual’s memory concerns when participants choose to share their MCI diagnosis, as illustrated below,

*“all my friends know about it (MCI diagnosis) and they are making allowances for me, they’re very good like that”* (Pierce et al., 2016 p.5)

This also infers a tolerability, allowing others to be accepting of the situation but may not mean acceptance. Several authors found the consequences of revealing memory concerns to others was invariably negative, leaving people irritated or angry with family and friends reactions, (Begum et al., 2013; Joosten-Weyn Banningh et al., 2008; Meilak et al., 2016; Nakano et al., 2012; Vandermorris et al., 2017). This is exemplified in the following statement from a female participant who had revealed her MCI diagnosis to her family.

“*I’m sorry I told them now, I’m not an invalid”* (Meilak et al., 2016 p.5)

This was in response to a family perceived as being overbearing in their offers of help. Reactions from friends can also, invalidate experiences of memory concerns. (Gomersall et al., 2017). This is illustrated by Gommersall who describes an 84-year-old participant recounting how her friend ‘roared with laughter’ at hearing that she had attended the memory clinic for MCI diagnosis.

## Discussion

In this scoping review we selected 24 papers which included the experiences of 453 people either diagnosed with MCI or subjective cognitive decline (SCD), and others who self-identified with concerns about their memory. The majority of participants living with memory concerns with or without a diagnosis of MCI/SCD were female, from White ethnic majority populations (or from studies where ethnicity was not identified) and with high levels of education. We identified two themes; *Making a diagnosis personal* and *Remembering not to forget*. We found that individuals understand cognitive concerns through normative comparison with others of the same age and this is associated with a range of responses including fear, relief, and acceptance but culminating in uncertainty.

Mild cognitive impairment and SCD do not have homogeneous underlying pathologies or a predictable disease course and the ability of a diagnosis to reassure and help people has been questioned, (Gifford et al., 2015; Howard, 2020; Pickersgill, 2014). This contributes to the feelings of uncertainty expressed within the literature. The concept of ‘manufactured uncertainty’, (Giddens, 1999) has resonance here where more knowledge does not lead to clarity but its opposite; consequently, the diagnostic process surrounding MCI rarely appears to produce certainly and understanding. Despite its significance in the prodromal phase of dementia, MCI remains problematic due to a lack of standardised criteria (Schweda et al., 2018). The contested nature of MCI is not only about clinical nosology but rather is about how people’s lives are not reducible to stages of a predefined disease progression. The lack of an explicit patient career creates not only uncertainty but also an existential vacuum. We found seemingly fatalistic responses with participants talking about ‘resigning’ to MCI. Individuals are forced to navigate a range of unclear meanings without any prospect of clarity or resolution.

Our scoping review identified that individuals normalise memory loss through comparison with peers of a similar age. As such individuals seek to gauge their own individual journey, in the absence of any clear ‘external’ trajectory following memory concerns and MCI. Unlike the more established concept of ‘biographical disruption’ where the diagnosis creates a new understandable narrative for the patient (Bury, 1982) conditions such as MCI do not lead to the same permanence. As Llewelyn points out, diagnosis should make a disease more navigable, ‘‘fixing the terrain over which care can be mapped’’ (Llewelyn H, 2017). However, in the case of memory concerns the terrain is not easily read and its status as a disease is questionable. Our findings reflect the features of a diagnosis of dementia noted by others; it rarely happens at a single point in time but, rather across time and is situated in the physical and social changes realised by the individual and others (Birt et al., 2017).

Our first theme ‘making a diagnosis personal’ captures the uncertainty surrounding reactions and responses to a diagnosis of MCI. Its supports the notion of a liminal experience for those living with MCI and reflects its contested nature. Birt et al., (2017) describes liminality as relating to people being pushed into an in-between state through a diagnosis which results in uncertainty and confusion. We found that as familiar roles altered through retirement and familial roles were compromised through cognitive changes, an individual’s sense of place and position in society was disrupted. Birt et al., (2017) points out that cognitive changes also mark a sociological event, as planned trajectories, roles and statuses are threatened. Unclear trajectories mean individuals find themselves in a sustained liminal state which Kelly (2010) describes as ‘learning to live with liminality’. However, contemporary descriptions challenge this focus on liminality, with notions of active or successful ageing challenging discourses of decline and deficit, (Birt et al., 2017). Birt et al talks about a post-liminal state as people reposition themselves through agentic actions which support social citizenship such as active ageing. (Birt et al., 2017). We can see from our findings that participants put a considerable amount of effort into actively responding to their memory concerns through prevention strategies.

Our second theme ‘remembering not to forget’ describes compensatory strategies people adopt in the face of memory concerns and how individuals navigate relationships with others. (Pierce et al., 2016) highlight that when people ‘make allowances’ for friends with memory concerns they are treated differently, drawing attention to notions of self-acceptance and seeking acceptance. Relationships can also be sources of stress and anxiety especially when familial roles change. Role transformation and re-negotiation of long-standing roles within dementia has been considered by (Fletcher, 2020) as the ‘dyadic career’ and relates to spousal dyads. Distinct caring roles in relationships MCI are not as defined as where an individual has dementia, however the relational aspect remains significant. Tanner, (2016) describes ‘relational agency’, as a way of preserving identity. Cognitive concerns is not simply a negation of agency but involves taking on of new forms of agency, exercised in new ways Tanner (2016). We found individuals navigated relationships with family and others as a way of exercising agency. How people place and position themselves in relation to others is significant and these relationships can create supportive or stressful encounters.

We found that the absence of knowing what to do after a diagnosis of MCI can create an urge to act. Within the culture of active ageing or ‘successful ageing’, this implies an expectation to undertake healthy interventions to slow cognitive deterioration following confirmation of memory impairment, with or without a diagnosis (Jones & Higgs, 2010). The notions of distinction identified in healthy ageing discourse describe how individuals prove they are actively resisting cognitive decline and as such distancing themselves from others, (Libert et al., 2020). Within this context societal expectations about age and ageing are predominately negative. Colliers et al. (2020) study discovered that concern around cognitive changes were secondary to the social and emotional changes associated with ageing in general. Understanding this through the concepts of the third and fourth age draws attention to the networks of social, material, and cultural resources which support ‘normative differences’ and away from ‘life stage identifiers’ in later life (Gilleard & Higgs, 2014). For these authors, the cultures of the third age are characterised by discourses of agency, choice, consumerism and the motivating desire to not be seen as old. In contradiction, the fourth age is conceived as a ‘social imaginary’ which projects the most negative aspects of decline in ageing onto society (Gilleard & Higgs, 2014). Within this social imaginary events such as ‘falling’ act as social markers of the pull of being defined by the fourth age. A diagnosis of MCI or concerns about one’s memory fall into this category and can be conceived as warnings of what may lie ahead.

MCI is accompanied by temporal factors. We found that people use timelines to position themselves in relation to others establishing their own trajectory. Trajectories don’t follow a linear progression but exist as a range of individual experiences defined by liminality but characterised through anxiety and unknowing. Individuals adopt a range of strategies in attempts to cope and stave off dementia. Sometimes this is an inaction, a desire to do nothing which appears at odds with current rhetoric about active ageing. Romaioli and Contarello (2019) argue that although considered ‘defeatist’ within contemporary successful or active ageing discourses, the absence of activism can be viewed as an active choice and offers another perspective to the fatalistic or acceptance attitudes we saw within our results, Romaioli and Contarello (2019) suggest that there is a risk that the idea of agency is defined as the capacity for active commitment and in this sense deprives the individual of the possibility of constructing a different relationship with time. Understanding this as a ‘living’ rather than ‘lived experience’, reflects and acknowledges individual’s attempts to navigate memory concerns and MCI which affirms self-esteem and agency, however that is exercised.

### Strengths and limitations

Multiple terms synonymous with memory problems exist, including various diagnostic terminology surrounding SCD and MCI. Therefore, decisions were made to include some search terms at the exclusion of others and as such may have restricted our breadth of search in this area. However, that aside, our search terms brought together a broad range of areas within a complex topic.

Our research questions included people with both a diagnosis of either MCI or SCD alongside those concerned about memory. We noted these distinctions but did not attempt to compare groups according to labels, given our conceptual understanding that the labels often reflected help-seeking rather than underlying pathology. Gaps in the literature reflect the subjective nuanced differences in people ascribed a diagnosis of MCI diagnosis and those who are not, including culturally diverse experiences which need to be considered in future research.

## Conclusion

We have explored individual subjective experiences of memory concern, with or without a diagnosis of MCI, and more broadly how this interacts with the notion of cognitive impairment and age. Normalised and understood through age, societal expectations around active ageing adds to the complexity of these experiences. Individuals must navigate their own narrative and trajectory through memory impairment in the hope of creating certainty and maintaining agency. While a minority of people living with memory concerns are ascribed a diagnostic label of SCD/MCI. Acknowledging this as experience of potential anxiety and unknowing informs thinking about MCI as a diagnostic a category and more readily encompasses the emotional and psychological impact of this on people lives. The uncertainty surrounding an MCI diagnosis and associated disconnect with a sense of self and agency is still concerning. It is difficult to know how this compares to the experiences of most people who live with memory concerns who have not sought help. These questions require future exploratory research and increased understanding of the emotional and lived experiences of people with MCI to be taken into account when delivering public services.

## Appendix 1

1 mild cognitive impairment.mp. (21,418)
2 MCI.mp. (20,925)
3 (mild cognitive impairment adj20 diagnos*).mp. [mp = title, abstract, original title, name of substance word, subject heading word, floating sub-heading word, keyword heading word, organism supplementary concept word, protocol supplementary concept word, rare disease supplementary concept word, unique identifier, synonyms] (3237)
4 subjective cognitive decline.mp. [mp = title, abstract, original title, name of substance word, subject heading word, floating sub-heading word, keyword heading word, organism supplementary concept word, protocol supplementary concept word, rare disease supplementary concept word, unique identifier, synonyms] (954)
5 SCD.mp. [mp = title, abstract, original title, name of substance word, subject heading word, floating sub-heading word, keyword heading word, organism supplementary concept word, protocol supplementary concept word, rare disease supplementary concept word, unique identifier, synonyms] (14,392)
6 pre dementia.mp. [mp = title, abstract, original title, name of substance word, subject heading word, floating sub-heading word, keyword heading word, organism supplementary concept word, protocol supplementary concept word, rare disease supplementary concept word, unique identifier, synonyms] (308)
7 memory concern*.mp. [mp = title, abstract, original title, name of substance word, subject heading word, floating sub-heading word, keyword heading word, organism supplementary concept word, protocol supplementary concept word, rare disease supplementary concept word, unique identifier, synonyms] (164)
8 memory complaint*.mp. [mp = title, abstract, original title, name of substance word, subject heading word, floating sub-heading word, keyword heading word, organism supplementary concept word, protocol supplementary concept word, rare disease supplementary concept word, unique identifier, synonyms] (1664)
9 memory loss.mp. [mp = title, abstract, original title, name of substance word, subject heading word, floating sub-heading word, keyword heading word, organism supplementary concept word, protocol supplementary concept word, rare disease supplementary concept word, unique identifier, synonyms] (5181)
10 cognitive decline.mp. [mp = title, abstract, original title, name of substance word, subject heading word, floating sub-heading word, keyword heading word, organism supplementary concept word, protocol supplementary concept word, rare disease supplementary concept word, unique identifier, synonyms] (28,324)
11 cognitive impairment.mp. [mp = title, abstract, original title, name of substance word, subject heading word, floating sub-heading word, keyword heading word, organism supplementary concept word, protocol supplementary concept word, rare disease supplementary concept word, unique identifier, synonyms] (72,318)
12 senior moment*.mp. [mp = title, abstract, original title, name of substance word, subject heading word, floating sub-heading word, keyword heading word, organism supplementary concept word, protocol supplementary concept word, rare disease supplementary concept word, unique identifier, synonyms] (17)
13 (ageing or aging).mp. [mp = title, abstract, original title, name of substance word, subject heading word, floating sub-heading word, keyword heading word, organism supplementary concept word, protocol supplementary concept word, rare disease supplementary concept word, unique identifier, synonyms] (420,018)
14 older people.mp. [mp = title, abstract, original title, name of substance word, subject heading word, floating sub-heading word, keyword heading word, organism supplementary concept word, protocol supplementary concept word, rare disease supplementary concept word, unique identifier, synonyms] (35,544)
15 elder*.mp. [mp = title, abstract, original title, name of substance word, subject heading word, floating sub-heading word, keyword heading word, organism supplementary concept word, protocol supplementary concept word, rare disease supplementary concept word, unique identifier, synonyms] (300,630)
16 older adult*.mp. [mp = title, abstract, original title, name of substance word, subject heading word, floating sub-heading word, keyword heading word, organism supplementary concept word, protocol supplementary concept word, rare disease supplementary concept word, unique identifier, synonyms] (103,399)
17 later life.mp. [mp = title, abstract, original title, name of substance word, subject heading word, floating sub-heading word, keyword heading word, organism supplementary concept word, protocol supplementary concept word, rare disease supplementary concept word, unique identifier, synonyms] (12,852)
18 old age.mp. [mp = title, abstract, original title, name of substance word, subject heading word, floating sub-heading word, keyword heading word, organism supplementary concept word, protocol supplementary concept word, rare disease supplementary concept word, unique identifier, synonyms] (31,872)
19 geriatric*.mp. [mp = title, abstract, original title, name of substance word, subject heading word, floating sub-heading word, keyword heading word, organism supplementary concept word, protocol supplementary concept word, rare disease supplementary concept word, unique identifier, synonyms] (114,100)
20 senior*.mp. [mp = title, abstract, original title, name of substance word, subject heading word, floating sub-heading word, keyword heading word, organism supplementary concept word, protocol supplementary concept word, rare disease supplementary concept word, unique identifier, synonyms] (47,495)
21 experience*.mp. [mp = title, abstract, original title, name of substance word, subject heading word, floating sub-heading word, keyword heading word, organism supplementary concept word, protocol supplementary concept word, rare disease supplementary concept word, unique identifier, synonyms] (1,240,016)
22 feel*.mp. [mp = title, abstract, original title, name of substance word, subject heading word, floating sub-heading word, keyword heading word, organism supplementary concept word, protocol supplementary concept word, rare disease supplementary concept word, unique identifier, synonyms] (109,766)
23 understand*.mp. [mp = title, abstract, original title, name of substance word, subject heading word, floating sub-heading word, keyword heading word, organism supplementary concept word, protocol supplementary concept word, rare disease supplementary concept word, unique identifier, synonyms] (1,396,650)
24 (perceive or perception*).mp. [mp = title, abstract, original title, name of substance word, subject heading word, floating sub-heading word, keyword heading word, organism supplementary concept word, protocol supplementary concept word, rare disease supplementary concept word, unique identifier, synonyms] (508,288)
25 attitude*.mp. [mp = title, abstract, original title, name of substance word, subject heading word, floating sub-heading word, keyword heading word, organism supplementary concept word, protocol supplementary concept word, rare disease supplementary concept word, unique identifier, synonyms] (462,207)
26 meaning*.mp. [mp = title, abstract, original title, name of substance word, subject heading word, floating sub-heading word, keyword heading word, organism supplementary concept word, protocol supplementary concept word, rare disease supplementary concept word, unique identifier, synonyms] (159,712)
27 perspective*.mp. [mp = title, abstract, original title, name of substance word, subject heading word, floating sub-heading word, keyword heading word, organism supplementary concept word, protocol supplementary concept word, rare disease supplementary concept word, unique identifier, synonyms] (394,061)
28 belief*.mp. [mp = title, abstract, original title, name of substance word, subject heading word, floating sub-heading word, keyword heading word, organism supplementary concept word, protocol supplementary concept word, rare disease supplementary concept word, unique identifier, synonyms] (95,944)
29 view*.mp. [mp = title, abstract, original title, name of substance word, subject heading word, floating sub-heading word, keyword heading word, organism supplementary concept word, protocol supplementary concept word, rare disease supplementary concept word, unique identifier, synonyms] (521,025)
30 1 or 2 or 3 or 4 or 5 or 6 or 7 or 8 or 9 or 10 or 11 or 12 (118,963)
31 13 or 14 or 15 or 16 or 17 or 18 or 19 or 20 (856,109)
32 21 or 22 or 23 or 24 or 25 or 26 or 27 or 28 or 29 (3,999,165)
33 30 and 31 and 32 (7134)

## Appendix 2

**Table:**

Author/Year/Country	Research aims	Sample population	Data collection	Data analysis	Key themes
Kruger TM, (2014) USA	To develop a framework for understanding how and why older adults respond to a diagnosis of MCI.	Purposively selected subsample n = 15 of older people diagnosed with MCI from a longitudinal study on aging at the National Institute on aging-funded Alzheimer’s disease centre, Sanders-Brown centre on aging, University of Kentucky Age range 64–91	In depth interviews and two survey.	Grounded theory	Three overlying themes. 1. Uncertainty 2. Temporal framing and 3. Interaction & role with others. Six influencing processes: 1. Beliefs about the cause of MCI, 2. Role of significant others, knowledge of MCI/dementia. 3. Knowledge of others with MCI/dementia, 4. Personality 5. Uncertainty associated with MCI.
Beard and Neary (2013) USA	To explore the subjective experiences of individuals diagnosed with MCI, how individuals make sense of this diagnosis and its potential psychosocial impact.	Sub sample n = 18 diagnosed with MCI taken from larger study, ACCESS (assessing the cultural characteristics of elders and the support Systems) Average age n = 76	In-depth interview & two focus groups,	Grounded theory and thematic analysis	Overarching themes; 1. Uncertainty concerning definitions of memory loss, MCI, and AD 2. Uncertainty around distinctions between normal ageing and dementia Four common themes; 1. Questions around whether their condition was a disease or not. 2. Struggling to define MCI 3. Distancing themselves from Alzheimer’s. 4. Grappling with the social implications of the diagnosis vis a vis Alzheimer’s.
Imhof et al., (2006) Switzerland	To explore everyday experience of healthy elderly people worried about forgetfulness	Sample n = 32 from memory clinic, diagnostic screening to rule out dementia, only criteria for inclusion was that participants self-identified as forgetful, mean age 73.7	In depth interviews approach, coding and thematic development SPPSS used.	Grounded theory	Forgetfulness in daily life conceptualized as ‘doing forgetfulness’ three themes 1. Reducing complexity; 2. Creating and maintaining routines. 3. Dealing with feelings of embarrassment and shame
Begum et al., (2013) UK	To explore beliefs, attitudes, and help-seeking behaviours in subjective cognitive decline and compare and contrast experiences.	Purposively selected sample n = 18 all-female; from a memory assessment service and a community survey; following MMSE all within a ‘conventional non-impaired’ range. n = 9 who sought formal help mean age 78 n = 9 who did not seek formal help mean age 74	Semi structured interviews	Thematic framework	Three themes identified as facilitators and barriers to formal health service use: 1. Concern, 2. Causation, 3. Perception of general practitioner. Two further areas shaping responses: 1. Informal help seeking and 2. Alternative pathways to care
Corner and Bond (2004) UK	To explore and further understand the perceptions of older people, who, because age are at risk of developing dementia.	Purposively selected sub-sample n = 15, from a wider study of quality of life from the perspective of older people with dementia and of their caregivers. Age range 62–93	Interviews using a topic guide based on literature and acting as a memory aid and refined as progressed.	Thematic framework.	Results found that participants felt; 1. Fear of dementia 2. Uncomfortable with friends or relatives with dementia. 3. Reluctance to contact health professionals about memory problems. 4. Uncertainty about the causes of dementia, 5. Anxieties about loss of self-identity and dignity, and long-term care. Key theme; older people without dementia avoid talking & thinking about dementia and do not plan for it.
Werner (2004) Israel	To explore elderly persons’ perceptions regarding their intentions to seek medical help,	A convenience sample n = 79 Taken from ‘community dwelling setting’ screened for subjective memory concern using CAMDEX (1988) Mean age 67.6	Semi-structured interviewing. Interview schedule developed to cover the health belief model (HBM) components.	Thematic analysis, HBM used as a conceptual framework	Results found participant beliefs and several influences act on willingness to seek memory assessment; 1. Memory problems inevitable part of aging, psychological consequences the main impact of these problems. 2. Seeking care presented as a result of fatalistic beliefs, ‘nothing can be done’.
Berg et al., (2013) Sweden	To explore experiences among individuals who have lived with MCI over 7 years without converting to dementia.	Sub-sample n = 17 recruited from the longitudinal Gothenburg MCI-study starting in 1999, mean age 72	Interviews at one single occasion.	Thematic analysis of the transcripts	Themes around life situations. Experiences of living with MCI status resulting in four themes: 1. At that time, when I came to the memory Clinic. 2. Adjusting to reduced capacity; 3. Worries about what is to come. 4. I have a good life.
De Vriendt et al., (2012) Belgium	To gain a deeper understanding of the functional decline in the process of MCI, they hypothesized that patients with MCI show subtle functional deficits in a-ADL and explored which a-ADL were impaired and to what extent.	Purposive sampling n = 37 from geriatric day hospitals all meeting MCI criteria. Age range 66–87	Semi-structured, in-depth interviews covered experiences of daily activities and changes in performance occurring over time.	Constant comparative analysis International classification of functioning, disability & health (ICF) (WHO, 2001), used to cluster activities	Results show participants diagnosed with MCI do not have performance problems in b-ADL and i-ADL. 1. Participants reported subtle problems in performance concerned with more complex a-ADL leisure, self-development. 2. Cyclic process of adaptation and coping mechanisms interacting with reduced skills can lead to emotional and functional consequences. 3. ADL important concept in the diagnosis of MCI.
Collier et al., (2020) New Zealand	To provoke discussion about how design processes and practices are embroiled in the construction of new medical diagnoses, through a web design project called ‘living well with MCI’ (2015)	Sub-sample n = 26 recruited from web design project and ethnographic study in memory clinics, n = 11 with MCI and n = 8 memory complaints An additional n = 7 family members Mean age 73.6.	Case study using interview data and recordings of discussions with anthropologist and web designer	Exploration of relationship between social, cultural, political and economic, contexts in which design processes unfold.	Results indicate that; 1. The website helped legitimize the MCI diagnosis by giving it form, despite ongoing debates about MCIs use and validity in clinical practice. 2. Older people (or ‘users’) internalized biomedical ideas as they negotiated what it meant to age ‘normally’ vs ‘abnormally’.
Dean et al.,(2014) UK	To investigate the experiences of people with mild cognitive impairment and their advocates. People with mild cognitive impairment (PWMCI)	Sample; n = 23 all diagnosed with MCI, recruited from research databases and memory clinics Age range 63–86 n = 20 advocates recruited Age range 42–84	Semi-structured interviews.	Qualitative analysis using methods based on grounded theory.	Results found that, 1. PWMCI rarely reported negative impressions of their GP. Positive impressions = finding the service to be “well run” Negative ones = assessment process or perceived lack of feedback. 2. Most PWMCI had no suggestions for improvements to their healthcare. 3. A variety of information was aimed at PWMCI.
Giebel et al., (2016) UK	To investigate how south asians with self-defined memory problems, with and without GP consultation, construe the symptoms, causes, consequences and treatment of the condition. It hypothesised that perceptions could vary depending upon consulting behaviour	Sample n = 33 recruited through community centres, networks & memory clinics all with self- defined memory problems. Group 1 n = 15 consulted GP Group 2 n = 18 no GP consultation Age 65+	Mixed methods study using the BEMI-D (Bart’s explanatory model inventory for dementia) and MMSE & GDS	Analysis through statistical tests chi square & t-test of the answers to BEMI-D but perceptions also recorded and noted.	Results; differences in perceptions linked to consulting practice, Group 1 non-consultation 1. Memory difficulties in terms of spiritual 2. Non active let thigs run, normalised 3. Increased co-morbidities and high depression rating using GDS but no formal diagnosis Group 2 consultation with GP 1. More proactive in action, 2. Invest in relationships, hearing & vision support access hospital treatment. 3. Increased diagnosis and treatment of depression. Understanding dementia depended upon whether a GP had been consulted.
Gomersall et al., (2017) UK	To understand the perceived benefits and drawbacks of a mild cognitive impairment (MCI) diagnosis and the drawbacks this diagnosis confers on individuals and their families.	Total sample n = 42 recruited from a memory service, (clinician identified people coded as having MCI in last 6 months) family members recruited for their perspective, MCI sample n = 18 Age range 60–93	Semi structured interviews using a topic guide.	Grounded theory approach was used.	Results found diagnosis benefits and draw backs. 1. Emotional impact of the diagnosis. 2. Practical benefits and limitations of the diagnosis, understanding symptoms, accessing clinical support. Participants glad of clinical support but frustrated at lack of clarity & lack of available treatments. Living with MCI is an ambivalent experience.
Hill et al., (2018) USA	To describe experiences of older adults living with subjective memory impairment (SMI) and examine the extent to which SMI severity was associated with impact of SMI on daily life	Sample n = 19 screened as having subjective memory impairment (SMI) Recruited from community setting in rural and urban Pennsylvania, mean age 80.7	A mixed methods convergent design. Semi-structured interviews	Thematic analysis.	Results found that; 1. Impact of SMI varied depending meanings individuals attributed to their experience. 2. The impact of memory problems ranged from frustration, embarrassment to avoidance of social activities. 3. Degree of emotional impact was not reflected in SMI severity or cognitive status.
Joosten-Weyn Banningh et al., (2008) Dutch	To investigate how patients fulfilling MCI criteria experience and cope with their cognitive decline. Secondary aim to derive key themes for MCI support group.	Purposive sample n = 8 patients with diagnosis of MCI recruited from the memory clinic of the Nijmegen University medical Centre. Age range 58–83	Qualitative approach using interviews and topic guide.	Grounded theory approach	Results found four themes: 1. Changes to cognitive abilities, mobility, affect, and somatic complaints. 2. Numerous attributions concerned aetiologies such as personality traits and overload of information. 3. Consequences all negative and concerned patients, such as anxiety, loss of self-confidence, feelings of anger towards others. 4. Patients applied emotion-oriented, problem-focused and avoidant coping strategies
Lingler et al., (2006) USA	To describe the subjective experience of living with and making sense of the diagnosis of mild cognitive impairment.	Sample n = 12 recruited patients amnestic MCI (n = 6) non-amnestic MCI (n = 6) from a university-based memory disorders clinic. Age range 65–86	Semi-structured interviews with people within 3–6 months of them receiving a diagnosis of the syndrome.	Grounded theory used to analyse the data,	Results; adjusting to a diagnosis of MCI, or assigning meaning, fundamental aspect of living. 1. This process comprised interrelated emotional and cognitive dimensions. 2. Expectations of normal aging, personal experience with dementia, and concurrent health problems were used to assign meaning to MCI diagnosis.
Nakano et al., (2012) Japan	To explore the subjective and emotional experiences of MCI of community dwelling older people and their family	Sample n = 2, recruited from ‘community dwelling’ both diagnosed with mild cognitive impairment (MCI) Ages 74 & 80	Semi structured interviews, asking the question ‘’please talk about the things that are troubling you’’	Qualitative inductive method	Results showed four categories. 1. Bewilderment regarding memory decline, 2. Avoidance of neighbourly relations, 3. Desire to maintain a healthy life 4. Psychological impact of familial relationship in constant fluctuation
Parikh et al., (2016) Canada	To explore the impact that mild memory changes can have on everyday lives. Older adults with age-normal memory changes and those with amnestic (aMCI).	Purposive sample n = 37 recruited and screened from Baycrest health Sciences Centre. Two groups; n = 23 ‘age-normal memory ability’ Age range 64–82 n = 14 aMCI (amnesic MCI) Age range 73–89	Qualitative research design, data collected from three focus groups	A thematic analysis using the constant comparative method.	Results found mild memory changes have a meaningful impact on several aspects of daily life; 1. Changes in feelings and views of the self, 2. Changes in relationships and social interactions, 3. Changes in work and leisure activities. 4. Deliberate increases in compensatory behaviours. These were more substantial and more adverse for individuals with aMCI than for those with age-normal memory changes.
Pierce et al., (2016) UK	To identify how people with a diagnosis of mild cognitive impairment used language in order to reveal the societal views and shared meanings of the diagnosis, and the positions taken by people.	Purposive sample n = 7 diagnosed with MCI recruited from memory clinics in North Wales, age range 60–78,	Interviews were conducted using a schedule.	Discourse analysis	Results revealed discourses around ‘Not knowing’ about MCI. Participants positioned themselves between two more familiar discourses; 1. ‘Knowing about ageing and dying’ and ‘Not wanting to know’ about dementia. 2. Consideration of where MCI is positioned in respect to normal ageing and dementia.
Choi and Kim (2020) South Korea	To understand how older adults with (MCI) perceived their condition following diagnosis and to explain the process of coping with concomitant changes in their lives.	Purposive sample n = 20 inclusion 1 year post diagnosis of MCI recruited through the head of a dementia support centre Age range 69–75	Theoretical sampling methods and in-depth interviews.	Grounded theory	Results; core category of living a daily life with self-awareness. Four stages; 1. Accepting their diagnosis 2. Strengthening their volition, 3. Taking care of their health 4. Maintaining a daily life with self-awareness, avoiding causing inconvenience to others
Rotenberg et al., (2020) Israel	To examine the lived experience of older adults who seek medical help for perceived memory problems,	Sub-sample n = 12 of larger quantitative study characterising ‘help-seekers’ from 4 geriatric clinics, n = 12 out of n = 51 recruited for qualitative study Inclusion; memory problems for at least 6 months. Age 65 upwards	Semi structured interviews.	Interpretative phenomenological analysis. Dedoose software	Results; three types of coping responses related to age context and social environment context: 1. Active problem solving, 2. Reframing perceptions of the problem 3. Avoidant behaviours, strategies to deal with memory problems and impact include four mechanisms; a) minimization, b) Normalization, c) rationalisation d) sense of control. Cognitive training alone will not make a meaningful change in lived experience.
Vandermorris et al., (2017) Canada	To gain in-depth insight into therapeutic mechanisms, benefits, and impact of a multimodal behavioural memory intervention for older adults concerned about memory.	Sub sample n = 11 of n = 53 recruited from memory and ageing programme all categorised as having ‘normal age memory decline’ Age range 63–88	Semi-structured interviews post-intervention.	Qualitative content analysis	Results; themes, normalization, benefit of participation, ‘accepting where I’m at’. 1. Both specific intervention content and process of participating with others. 2. A positive impact; emotional; (feelings of reassurance, hope, and confidence). Functional (increasing motivation for lifestyle change)
Meilak et al., (2016) UK	Examine older people’s understanding of MCI, explore attitudes towards disclosure of a hypothetical diagnosis of MCI and the experience of receiving a diagnosis of MCI.	Purposive sample n = 10 recruited from two memory clinics in London Two groups; n = 7 ‘cognitively intact’ n = 6 MCI diagnosis Age 65 upwards	Semi structure interviews Group one; with hypothetical vignette of MCI Group two; exploring thoughts around diagnosis	Thematic analysis using Nvivo 10	Results; MCI group themes; • Understanding of MCI • Concerns regarding memory loss • Consequences of getting a diagnosis • Coping strategies
Renn et al., (2021) USA	To explore subjective experience of a “typical week” living with MCI to document (a) important activities, (b) barriers to usual activities, and (c) facilitators and supports	Sample n = 11 recruited from a University of Washington Harborview medical centre memory & brain Wellness centre research register & self through flyers Included if diagnosis of MCI Age range 57–79	Interviews and use of photographs	Thematic analysis and photo elicitation	Three themes: (a) important activities in a typical week, (b) disruptions to a typical week, (c) facilitators or compensatory supports. Two unexpected themes; (d) disclosure of diagnosis (e) reflections about the future.
Poppe, et al., (2020) London UK	To explore how people with MCI or SCD and other stakeholders consider how future dementia prevention (AT programme) should be designed for and delivered to this group.	Purposive sample n = 18 with memory concerns recruited from NHS memory clinics, primary care, IAPT; UCL/third sector organisations age 60 upwards	Qualitative interviews	Thematic analysis Nvivo 12	Three themes: (1) acknowledging the liminal state, (2) enabling change in challenging contexts, (3) building on existing values, cultures, and routines

## Appendix 3

**Table:**

Key----- = This was unclear x = Not apparent + = Yes was evident	Clear statement of research aims	Qualitative methodology appropriate	Appropriate research design	Appropriate recruitment strategy	Appropriate data collection	Researcher and participant relationship considered	Ethical issues considered	Sufficiently rigorous data analysis	Finding clearly stated	Valuable research
(Kruger TM, 2014)	**+**	**+**	**+**	**+**	**+**	**----**	**+**	**+**	**+**	**+**
(Beard & Neary, 2013)	**+**	**+**	**+**	**+**	**+**	**----**	**x**	**+**	**+**	**+**
(Imhof et al., 2006)	**+**	**+**	**+**	**+**	**+**	**x**	**+**	**+**	**+**	**+**
(Begum et al., 2013)	**+**	**+**	**+**	**+**	**+**	**+**	**+**	**+**	**+**	**+**
(Corner & Bond, 2004)	**+**	**+**	**+**	**+**	**+**	**x**	**x**	**----**	**+**	**+**
(Werner, 2004)	**+**	**+**	**----**	**+**	**+**	**----**	**x**	**----**	**+**	**----**
(Berg et al., 2013)	**+**	**+**	**+**	**+**	**+**	**+**	**+**	**+**	**+**	**+**
(De Vriendt et al., 2012)	**+**	**+**	**+**	**+**	**+**	**----**	**+**	**+**	**+**	**+**
(Collier et al., 2020)	**+**	**+**	**+**	**+**	**+**	**+**	**+**	**----**	**+**	**+**
(Dean et al., 2014)	**+**	**+**	**+**	**+**	**+**	**x**	**+**	**+**	**+**	**+**
(Giebel et al., 2016)	**+**	**+**	**+**	**+**	**+**	**----**	**----**	**+**	**+**	**+**
(Gomersall et al., 2017)	**+**	**+**	**+**	**+**	**+**	**+**	**+**	**+**	**+**	**+**
(Hill et al., 2018)	**+**	**+**	**+**	**+**	**+**	**+**	**----**	**+**	**+**	**+**
(Joosten-Weyn Banningh et al., 2008)	**+**	**+**	**+**	**+**	**+**	**x**	**----**	**+**	**+**	**+**
(Lingler et al., 2006)	**+**	**+**	**+**	**+**	**+**	**+**	**+**	**+**	**+**	**+**
(Nakano et al., 2012)	**+**	**+**	**----**	**----**	**x**	**+**	**+**	**----**	**+**	**+**
(Parikh et al., 2016)	**+**	**+**	**+**	**+**	**+**	**+**	**+**	**+**	**+**	**+**
(Pierce et al., 2016)	**+**	**+**	**+**	**+**	**+**	**x**	**+**	**+**	**+**	**+**
(Choi & Kim, 2020)	**+**	**+**	**+**	**+**	**+**	**x**	**+**	**+**	**----**	**----**
(Rotenberg et al., 2020)	**+**	**+**	**+**	**+**	**+**	**----**	**+**	**+**	**+**	**+**
(Vandermorris et al., 2017)	**+**	**+**	**+**	**+**	**+**	**+**	**----**	**+**	**+**	**+**
(Meilak et al., 2016)	**+**	**+**	**+**	**+**	**+**	**----**	**X**	**+**	**+**	**+**
(Renn et al., 2021)	**+**	**+**	**+**	**+**	**+**	**+**	**----**	**+**	**+**	**+**
(Poppe et al., 2020)	**+**	**+**	**+**	**+**	**+**	**----**	**----**	**+**	**+**	**+**

**Key -----** = This was unclear **x** = This was not apparent **+** = Yes this was evident.
